# Fibroblast growth factor receptor 1 gene mutation as a potential risk factor for spontaneous intracranial hemorrhage in pediatric low-grade glioma patients

**DOI:** 10.1093/noajnl/vdae074

**Published:** 2024-06-04

**Authors:** Maxine Gonzalez-Vega, Brittany M. Lebert, Stephani Campion, Aaron Wagner, Ana Aguilar-Bonilla, Amy A. Smith

**Affiliations:** Department of Pediatrics, Division of Pediatric Hematology/Oncology, Neuro-Oncology Translational Lab, Orlando Health – Arnold Palmer Hospital, Orlando, Florida, USA; Department of Pediatrics, Division of Pediatric Hematology/Oncology, Neuro-Oncology Translational Lab, Orlando Health – Arnold Palmer Hospital, Orlando, Florida, USA; Department of Administration and Quality, Orlando Health - Orlando Health Advanced Rehabilitation Institute, Ocoee, Florida, USA; Department of Pathology, Orlando Health - Orlando Regional Medical Center, Orlando, Florida, USA; Department of Pediatrics, Division of Pediatric Hematology/Oncology, Neuro-Oncology Translational Lab, Orlando Health – Arnold Palmer Hospital, Orlando, Florida, USA; Department of Pediatric Hematology Oncology, Orlando Health – Arnold Palmer Hospital, Orlando, Florida, USA; Department of Pediatrics, Division of Pediatric Hematology/Oncology, Neuro-Oncology Translational Lab, Orlando Health – Arnold Palmer Hospital, Orlando, Florida, USA; Department of Pediatric Hematology Oncology, Orlando Health – Arnold Palmer Hospital, Orlando, Florida, USA

**Keywords:** FGFR1, LGG, PTPN11, spontaneous hemorrhage

## Abstract

**Background:**

Fibroblast growth factor receptor 1 (*FGFR1*) mutations have been associated with poorer prognoses in pediatric central nervous system tumor patients. A recent study highlighted a link between *FGFR1* mutations and spontaneous intracranial hemorrhage (ICH), demonstrating that all patients with an *FGFR1* alteration experienced hemorrhage at some point during their course of treatment.

**Methods:**

The current study examined 50 out of 67 pediatric patients with low-grade gliomas (LGGs) who had genomic testing between 2011 and 2022 at our institution to determine whether a correlation exists between *FGFR1* mutations and spontaneous ICH.

**Results:**

We found that of the 50 patients with genomic data, 7 (14%) experienced ICH, and an additional spontaneous hemorrhage was recorded; however, no genomic testing was performed for this case. Five of the seven patients (71.4%) had an *FGFR1* modification. In our patient population, 6 expressed a detectable *FGFR1* mutation (66.7% [4/6] had N546K alteration, 16.7% [1/6] *FGFR1* exons duplication, and 16.7% [1/6] had a variant of unknown significance [VUS]). The patient with the *FGFR1* VUS had no reported spontaneous hemorrhage. Statistical analysis found a significant association between *FGFR1* and spontaneous intracranial hemorrhage (*P*-value = < .0001). In the patient population, all cases of *PTPN11* alterations (*n* = 3) co-occurred with *FGFR1* mutations.

**Conclusions:**

Our case series highlights this link between the FGFR1 mutation and spontaneous intracranial hemorrhage in pediatric LGGs.

**Key Points**

- *PTPN11* alterations co-occur with *FGFR1* mutations in pediatric and young adult low-grade gliomas (LGGs).
- The present case series further supports the link between *FGFR1* mutations and spontaneous intracranial hemorrhage in pediatric patients with LGG.

**Importance of the Study**

For pediatric low-grade gliomas (pLGGs), *FGFR1* gene alterations have been linked to poorer prognosis and have been correlated with the manifestation of intracranial hemorrhage (ICH) in one reported cohort of patients. The study reports a statistically significant relationship between spontaneous hemorrhage in pediatric patients with *FGFR1*-altered LGG. The genomic profiles show a concurrent expression of *PTPN11* alteration with *FGFR1*, where all the cases exhibited ICH. The following clinical case series strengthens the link between FGFR1 alterations and spontaneous ICH.

Tumors of the central nervous system (CNS) are the second most common cancer in children and remain the leading cause of cancer-associated mortality in pediatric populations.^1^ Of the diverse types of pediatric CNS tumors, low-grade gliomas (LGGs) are the most common and comprise nearly one-third of all cases.^2^ Overall 5-year survival (OS) for children with LGGs is excellent even with incomplete tumor resection.^3^ However, these children require treatment with chemotherapies, radiation, and/or additional surgeries all of which are associated with morbidities that significantly impact the quality of life for patients, including long-term effects on the endocrine system and neurodevelopment.^4^

Pediatric LGGs are associated with genetic alterations in the mitogen-activated protein kinase (MAPK) signaling pathway.^3^ Most frequently, they harbor mutations within the pathway at the *Braf proto-oncogene* (*BRAF*) gene, specifically the *BRAF* V600E missense mutation and the *KIAA1549:BRAF* fusion.^4^ The second most common class of genetic alterations in pediatric LGGs occurs at the *fibroblast growth factor receptor 1* (*FGFR1*) gene, believed to drive the proliferation of multipotent stem cells in developing human brains.^2,5^*FGFR1* alterations, including the *FGFR1* N546K and K656E missense mutations, constitutively activate this pathway.^6^ In several pediatric CNS tumor types, including pilocytic astrocytoma (PA)—the most common type of low-grade glioma, *FGFR1* mutations have been linked to poorer prognoses.^7^

A recent study suggested an association between *FGFR1* mutations and spontaneous intracranial hemorrhage (ICH) in pediatric LGG patients.^8^ Although rare, intracranial hemorrhage can have devastating effects on pediatric populations. An estimated one-third of pediatric patients who experienced an ICH die,^9^ and those that survive may suffer neurodevelopmental effects^10^ from long-standing neurologic, cognitive, and adaptive behavior impairments affecting their potential independence and social functioning.^11^ In the aforementioned study, Ishi et al. found that all reported patients (*n* = 4) with an *FGFR1* mutation experienced spontaneous ICH at some point during their course of treatment.^8^ The significance of this finding could be highly impactful, as tumor-specific risk factors for spontaneous intracranial hemorrhage have yet to be identified.^8^ However, more studies with additional patients harboring *FGFR1* mutations are needed to further confirm these findings.

In the present study, we aim to contribute by presenting data from 50 patients treated for LGGs at our institution during 2011–2022 that had genomic testing performed. We investigate the frequency of spontaneous intracranial hemorrhage and link these events to their genetic mutations, specifically looking for any statistically significant correlation between hemorrhage and *FGFR1* mutations. Furthermore, we present any cases in which a patient with an *FGFR1* mutation experienced spontaneous intracranial hemorrhage, with the hopes of contributing to the current literature on this potential novel risk factor in pediatric LGG patients.

## Materials and Methods

### Patient Population

For this retrospective study, we included 67 pediatric cases from patients diagnosed and treated for LGGs at the Orlando Health Arnold Palmer Hospital for children from 2011 to 2022. The selection of the patients depended on their age (≤18 years) and the diagnostic grade for LGGs (grades I and II), previously used by the World Health Organization (WHO) to classify malignancies. Patient information accessed for this study included treatment courses, clinical outcomes, and radiographic and pathological findings. A secondary filtering was performed for patients who underwent genetic and molecular analyses at the time of tumor resection and biopsy. Fifty patients who fit the criteria remained and each had one or a combination of the following molecular analyses: Neuro-Oncology Expanded Gene Panel, Chromosomal Microarray, Methylation, and Next-Generation Sequencing. All patients examined in this study were consented under the IRB-approved Translational Research Protocol.

### Statistical Analysis

The statistical Fisher’s exact 2-sided test followed by the Baptista-Pike odds ratio method was performed with GraphPad Prism version 9.4.1 (GraphPad Software). A *P*-value < .05 was considered statistically significant.

## Results

### Genetic Mutations and Spontaneous Hemorrhage in Low-Grade Gliomas

Of the 50 patients with pediatric LGGs, only 6 cases have an *FGFR1* mutation (12%). However, 2 of the 6 patients do not exhibit the typical *FGFR1* point mutations, N546K or K656E, but present with either a duplication of exons 9–18 or a variant of unknown significance (VUS), R207H. The patient with a VUS also had a *KIAA1549:BRAF* fusion and did not present with an intracranial hemorrhagic stroke (case not discussed). Three out of fifty patients (6%) have *PTPN11* mutations (N58D, E69K, and E76K), all co-occurring with *FGFR1*. Twenty-seven of the fifty (54%) patients have a *BRAF* gene alteration (28% have the *KIAA1549* fusion, 20% have the *BRAF* V600E mutation, 4% have the *PRKARB2B* fusion, and 2% present with a *BRAF* duplication). Seven patients (14%) underwent molecular analysis, but no pathogenic mutations were detected. The remaining 11 patients (22%) had other mutations not discussed in this case study (*BRCA1, ERCA2-RAF1, IDH1, KRAS, MEK2, PIK3R1, RAF1, SDHC, SETBP1*, and *TERT*).

Of the 50 patients, 7 (14%) of them experienced spontaneous intracranial hemorrhage. Notably, 5 (83.3%) of the 6 patients with an *FGFR1* mutation manifested a spontaneous hemorrhage at some point during their course of treatment. The remaining 2 patients with spontaneous hemorrhage expressed either a *BRAF* V600E mutation or a *PRKAR2B:BRAF* fusion. Due to the frequency and interest of the *FGFR1* mutation in spontaneous hemorrhage cases, we present cases 1 through 5 where we observed the co-occurrence of the events (Table 1).

**Table 1.** Summary of Pediatric LGG Patients Presenting Spontaneous Hemorrhage

Case	Sex	Age at onset	Anatomical site	Pathology	Genetic mutation
1	M	17 years	Optic pathway	PA	**FGFR1 p.N546K** NF1 c.1721 + 2T > C PTPN11 p.N58D*
2	F	10 years	Optic pathway	PA	**FGFR1 p.N546K** PTPN11 p.E69K*
3	M	12 years	Hypothalamus	PA	**FGFR1 p.N546K** KRAS p.G12D
4	M	14 years	Suprasellar	PA	**FGFR1 p.N546K** PTPN11 p.E76K*
5	F	17 months	Optic pathway	PA	FGFR1 Dup.Exon9-18*
6	F	10 years	Left parietal	Glioneuronal	BRAF V600E
7	F	12 years	Left temporal	PA	PRKAR2B:BRAF Fusion*

PA, pilocytic astrocytoma; *patient presents with variants of uncertain significance, not reported here.

## Case Presentations

### Case 1

The male patient originally presented at age 9 with decreased vision and a history of Noonan’s Syndrome. Initial MRI imaging detected an optic pathway glioma, and the patient was started immediately on Carboplatin and Vincristine as per the Children’s Cancer Group (CCG)-A9952 regime A. He transferred to our institution the following year and continued treatment until MRI showed disease progression. He underwent a biopsy confirming the diagnosis as a PA and started a weekly Vinblastine treatment. Three months later, MRI again showed tumor progression and he underwent tumor debulking and cyst fenestration. He was started on Avastin and Everolimus for 12 cycles and continued Avastin due to tumor response. However, treatment was stopped due to intratumoral hemorrhage. He then received 14 cycles of Everolimus and 10 cycles of Carboplatin. Six months routine MRI again showed progression and the patient was treated with Trametinib, which he tolerated for 11 months but was then stopped due to a shunt revision.

**Figure 1.**
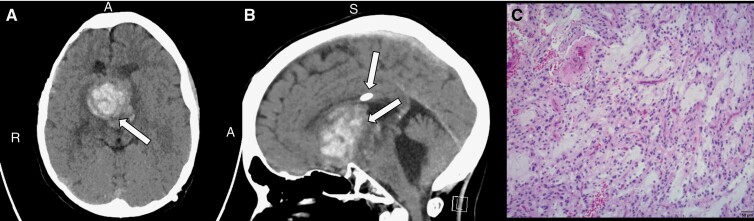
Case 1 brain CT scans without contrast show an intratumoral hemorrhage in the third ventricle. (A) Axial view of the brain with an arrow indicating the intratumoral hemorrhage. (B) Sagittal view of the brain with arrows indicating a shunt port (top) and the intratumoral hemorrhage with enlargement of the third ventricle (bottom). (C) Hematoxylin and eosin (H&E) stained slide image depicting a low-grade spindle cell neoplasm with alternating hypercellular and hypocellular areas (characteristic of a Pilocytic Astrocytoma). *A, Anterior; S, Superior; R, Right.* Scale bar = 50 µm.

At age 16, the patient underwent a second sub-total resection and molecular sequencing with reported positive mutations for *FGFR1*, *MEK2*, *PTPN11,* and *NF1* genes. The patient was diagnosed with progressive disease 3 months after surgery and treated under the NCI-Children’s Oncology Group (COG) MATCH Study with Erdafitinib, an *FGFR1* inhibitor, which initiated tumor response but was not tolerated by the patient. Due to his previous response, Avastin was restarted, but he again developed a large intracranial hemorrhage that made his tumor appear 3 times the size (Figure 1). He underwent a third partial resection followed by partial radiation, which was stopped due to another small hemorrhage. A decision was made to stop treatment, and the patient succumbed to his disease at the age of 18.

### Case 2

A female patient was diagnosed at age 5 with an optic pathway glioma. She underwent a biopsy and pathology analysis was consistent with a PA. She began treatment with Vincristine, Carboplatin, and Temozolomide as per Children’s Oncology Group (COG)-ACNS0223. MRI showed progressive disease 2 years later and she was started on monthly Temodar for a year. The following year, MRI once again showed progression and she was started on Avastin and Irinotecan. Once again, a year later progression was detected, and the patient underwent MRI-guided laser ablation of the tumor. After several months, she came to the ER unconscious and exhibited intracranial hemorrhage and cardiac arrest. At age 14, she transferred to our institution, where her tumor showed progressive disease and had a 40% increase in size. Her tumor tissue was sent for molecular testing and 2 mutations were detected in the *FGFR1* and *PTPN11* genes. Based on these findings the patient was started on Everolimus for 12 cycles, but once again the patient showed progressive disease. She was then started on Trametinib but switched to Vinblastine due to toxicity. She recently finished her treatment with Vinblastine and currently has stable disease.

### Case 3

**Figure 2.**
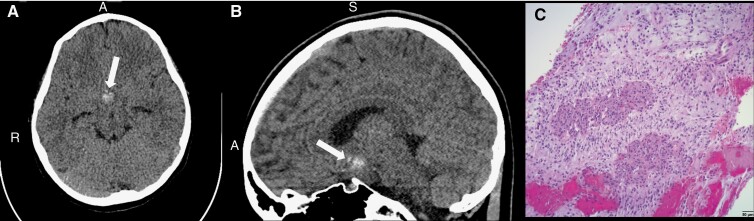
Case 3 brain CT scan without contrast showing an intratumoral hemorrhage extending to the fourth ventricular infundibular recess. (A) Axial view of the brain with an arrow indicating the intratumoral hemorrhage. (B) Sagittal view of the brain with an arrow indicating the fourth ventricular infundibular recess hemorrhage closer to the brain stem region. (C) At low magnification, hematoxylin and eosin (H&E) stained slide images depicting a low-grade spindle cell neoplasm with alternating hypercellular and hypocellular areas (characteristic of a pilocytic astrocytoma). *A, Anterior; S, Superior; R, Right.* Scale bar = 50 µm.

The male patient originally presented at age 8 with precocious puberty. MRI found a pituitary lesion that was thought to be a microadenoma, which was followed without changes. Three years later the patient had a sudden onset of headaches and emesis. MRI found a hemorrhagic suprasellar mass (Figure 2). He was referred to our institution, where he underwent a partial resection of the tumor. Pathology was consistent with a low-grade glioma, which was confirmed through molecular testing. Next-generation sequencing found that the tumor harbors an *FGFR1* and a *RAS* mutation. He was started on Carboplatin and Vincristine as per CCG-A9952A, and he is currently on cycle 8 with stable disease.

### Case 4

**Figure 3.**
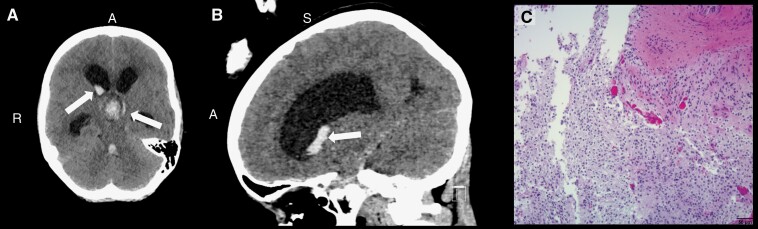
Case 4 brain CT scans without contrast showing an intratumoral hemorrhage. (A) Axial view of the brain with arrows indicating the intratumoral hemorrhage. (B) Sagittal view of the manifested intratumoral hemorrhage (arrow). (C) Hematoxylin and eosin (H&E) stained slide image at low magnification depicting a low-grade spindle cell neoplasm with alternating hypercellular and hypocellular areas (characteristic of a pilocytic astrocytoma). *A, Anterior; S, Superior; R, Right.* Scale bar = 50 µm.

Male patient presented at age 14 with acute headaches and an altered mental status. CT scan showed a suprasellar mass with intratumoral hemorrhage, as well as intraventricular hemorrhage (Figure 3). He underwent a biopsy, and the pathology was consistent with PA with *FGFR1* (p.N546K) and *PTPN11* mutations. MRI revealed leptomeningeal disease. He was treated with Carboplatin and Vinblastine as per protocol COG-ADVL0515. After 4 months of treatment, he had progressive disease and was started on Carboplatin, Vincristine, and Temozolomide as per COG-ACNS0223. Due to behavioral concerns, therapy was changed to CCG-A9952, omitting Vincristine due to neuropathy. The patient completed 8 cycles of therapy and had stable disease for 20 months. He then progressed and was treated with Everolimus for 9 months until the MRI again showed progressive disease. He was started on single-agent Temozolomide (200 mg/m^2^ days 1–5 every 28 days), but progressive leptomeningeal disease (LMD) was observed 6 months later. The patient is currently on therapy with Carboplatin and Temozolomide as per COG-ACNS0223.

### Case 5

**Figure 4.**
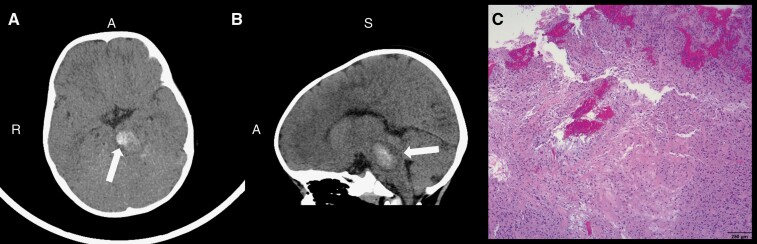
Case 5 brain CT scan without contrasts showing an intratumoral hemorrhage of the midbrain/brainstem. (A) Axial view of the brain with an arrow indicating the intratumoral hemorrhage. (B) Sagittal view of the brain with an arrow indicating the intratumoral hemorrhage. (C) At low magnification, the hematoxylin and eosin (H&E) stained slide image depicts a low-grade spindle cell neoplasm with alternating hypercellular and hypocellular areas (characteristics of a pilocytic astrocytoma). *A, Anterior; S, Superior; R, Right.* Scale bar, 250 µm.

Female patient presented at almost 1.5 years old with an acute midbrain hemorrhage that was initially thought to be a cavernoma (Figure 4). After the hemorrhage resolved, she was diagnosed with a brain tumor. The patient underwent 2 biopsies, though both were insufficient for diagnosis. That same year, she transferred to our institution after declining neurologically and developing hydrocephalus, where she underwent tumor debulking. Pathology was consistent with PA of the midbrain/pons and cerebellum, and molecular sequencing of the tumor revealed an *FGFR1* mutation. The patient was initially treated with Carboplatin and Vinblastine but was switched after 1 cycle to a weekly Carboplatin regimen per CCG-A9952 regimen A (without Vincristine) and completed 8 cycles.

After 19 months, she was found to have progressive disease, and treatment was restarted as per CCG-A9952 Carboplatin with desensitization protocol; however, further doses were held due to safety concerns. She was started on weekly Vinblastine for 2–12-week cycles, then every 2 weeks for 2–12-week cycles for 1 year of treatment. Avastin was added later every other week. Several months later, MRI again revealed progression, and treatment was changed to oral Trametinib, which she took for 7 months until progression was again noted. Her treatment was then changed to oral Temodar. Three months later she again progressed, and her treatment was changed to Trametinib and Everolimus.

### Pediatric LGGs Presenting Spontaneous Hemorrhage and the Factors Involved

The patient population has a stable age distribution for diagnosis and clinical presentation. Additionally, spontaneous hemorrhage in the study’s population does not favor a specific tumor location such as the optic pathway, brain stem, temporal lobe, parietal lobe, suprasellar region, and diencephalic region (*P*-value = .089, .370, > .999, .370, .263, and .140, respectively). Additionally, spontaneous hemorrhage shows no correlation with tumor pathology in the patient population of this study. Although the highest occurring mutation reported in this population involves *BRAF* alterations (27/50, 54%), there is no correlation between a disease-provoked intratumoral hemorrhage and *BRAF*. In contrast, both *FGFR1* and *PTPN11* gene alterations have correlated with spontaneous hemorrhage (*P*-value= < .0001, and .0018; Table 2). Interestingly, the *PTPN11* mutation exclusively occurred as a co-mutation with *FGFR1* in all the reported cases in this study.

**Table 2.** Univariate Analysis of Associations With Spontaneous Hemorrhage of the Patient Population With Genomic Data Using Fisher’s Exact Test

Factor	OR	95% Cl	*P*-value
Age			
<13 years.	1.588	0.185 to 20.06	>.999
Location			
Optic pathway	8.200	1.028 to 57.11	.089
Brain stem	3.417	0.204 to 31.85	.370
Temporal lobe	0.630	0.050 to 5.395	>.999
Parietal lobe	3.417	0.204 to 31.85	.370
Suprasellar region	7.000	0.318 to 135.0	.263
Diencephalic region	*+Inf*	0.698 to *Inf*	.137
Pathology			
PA/PMA	5.478	0.730 to 65.47	.124
Glioneuronal	3.500	0.209 to 32.60	.364
Genetic alteration			
* FGFR1*	220.0	12.84 to 2542	*<.0001*
* PTPN11*	**+*Inf*	6.878 to *Inf*	*.0018*
P-B fusion	7.167	0.326 to 138.2	.258
* BRAF* V600E	0.648	0.051 to 5.541	>.999

PA, pilocytic astrocytoma; PMA, pilomyxoid astrocytoma; Inf, infinite; P-B Fusion, PRKAR2B:BRAF Fusion.

Italics type *P*-value indicates statistical significance (*P* < .05).

## Discussion

To date, tumor-specific risk factors for spontaneous intracranial hemorrhage in pediatric LGGs have yet to be identified. Ishi et al., (2020) proposed a link between the *FGFR1* mutation and spontaneous hemorrhage events after they determined that all their patients with an *FGFR1* mutation (*n* = 4) experienced hemorrhage at some point during their course of treatment. *FGFR1* mutations are the second most altered gene in pLGGs and occur in 5%–10% of pLGG cases.^2^ In our patient population, 6 exhibited a detectable *FGFR1* alteration (12%). Four of the six (67%) reported cases detected one of the key *FGFR1* alterations, N546K, which have been associated with constitutive activation of the mitogen-activated protein kinase pathway.^12–14^ Only 2 of the 6 *FGFR1*-altered patients did not have any of the common alterations and instead expressed a duplication associated with increased activation of the pathway^15^ or a VUS that has yet to be regarded as pathogenic. The one patient with the VUS was the only *FGFR1* mutant with no reported spontaneous hemorrhage. Ishi et al., (2020) found a statistically significant correlation between the *FGFR1* alterations and these spontaneous intracranial hemorrhagic (ICH) events. Their findings favor the *FGFR1* K656E mutation, 3 out of the 4 patients (75%), and 1 of the patients (25%) presented with the N546K alteration. Correspondingly, the present study found that 4 out of the 5 cases (80%) with both ICH and FGFR1 mutants had the N546K alteration, with the extra case not being a point mutation but exons 9–18 duplication. Additionally, a statistical significance was established between intracranial hemorrhage and the *PTPN11* gene, which often co-occurs with *FGFR1*, suggesting a cooperative role of these mutations.^16^ Unlike Ishi et al., (2020), who found a statistical significance between hemorrhagic events and the diencephalic region, we did not see any correlations between intracranial hemorrhage and the location of the tumor.

Although there is currently no biological explanation for the association between *FGFR1* mutations and spontaneous hemorrhage, the cases highlighted by Ishi et al., our current study, and other case studies^17^ illustrating this link warrant the need for future investigation into the potential mechanisms behind it. Both *FGFR1* and *FGFR2* have been reported to be critical embryonic developmental factors, crucial for survival and injury response.^18^ Defects in the FGF/FGFR signaling pathway have been closely correlated with cardiovascular diseases due to the modulation of angiogenic and neovascularization responses.^19^ Furthermore, a tumor cancer manifestation in the CNS can be cataloged as an injury, meaning a vascular response occurs in many of the cases and an impaired FGF/FGFR signaling pathway such as a point mutation on the gene will affect the maintenance of neovascular vessels. Additional case studies with a larger number of patients are needed to directly link *FGFR1* mutations and spontaneous hemorrhage in pLGGs.

## Conclusion

Although uncommon, spontaneous intracranial hemorrhage can have devastating effects on pediatric low-grade glioma patients. To date, little is known about the tumor-related risk factors for these hemorrhagic events. However, a recent study proposing the *FGFR1* mutation as a potential novel risk factor for spontaneous intracranial hemorrhage in pLGG patients warrants further studies. The current study is consistent with their results, giving weight to their findings and validating the need for additional investigation to discover the specific mechanisms behind the association. This knowledge could help clinicians better predict the occurrence of spontaneous hemorrhages and provide further insight into the biology of these tumors to lead us toward continued improvement in our targeted treatments.

## Data Availability

No new data was generated in support of this research.
